# Zap1 Regulates Zinc Homeostasis and Modulates Virulence in *Cryptococcus gattii*

**DOI:** 10.1371/journal.pone.0043773

**Published:** 2012-08-20

**Authors:** Rafael de Oliveira Schneider, Natully de Souza Süffert Fogaça, Lívia Kmetzsch, Augusto Schrank, Marilene Henning Vainstein, Charley Christian Staats

**Affiliations:** 1 Centro de Biotecnologia, Universidade Federal do Rio Grande do Sul, Porto Alegre, Brazil; 2 Departamento de Biologia Molecular e Biotecnologia, Universidade Federal do Rio Grande do Sul, Porto Alegre, Brazil; University of Minnesota, United States of America

## Abstract

Zinc homeostasis is essential for fungal growth, as this metal is a critical structural component of several proteins, including transcription factors. The fungal pathogen *Cryptococcus gattii* obtains zinc from the stringent zinc-limiting milieu of the host during the infection process. To characterize the zinc metabolism in *C. gattii* and its relationship to fungal virulence, the zinc finger protein Zap1 was functionally characterized. The *C. gattii ZAP1* gene is an ortholog of the master regulatory genes *zafA* and *ZAP1* that are found in *Aspergillus fumigatus* and *Saccharomyces cerevisiae*, respectively. There is some evidence to support an association between Zap1 and zinc metabolism in *C. gattii*: (i) *ZAP1* expression is highly induced during zinc deprivation, (ii) *ZAP1* knockouts demonstrate impaired growth in zinc-limiting conditions, (iii) Zap1 regulates the expression of *ZIP* zinc transporters and distinct zinc-binding proteins and (iv) Zap1 regulates the labile pool of intracellular zinc. In addition, the deletion of *ZAP1* reduces *C. gattii* virulence in a murine model of cryptococcosis infection. Based on these observations, we postulate that proper zinc metabolism plays a crucial role in cryptococcal virulence.

## Introduction

The function of many proteins depends upon the essential role of Zn, acting as both a catalytic constituent and as a core component of structural motifs. The zinc metalloproteome consists of more than 300 proteins in yeast, and the majority of these proteins are zinc finger transcription factors [1]. The molecular mechanisms that control zinc homeostasis in cells are best characterized in *Saccharomyces cerevisiae*. The critical importance of zinc in yeast cells is suggested by the fact that approximately 3% of the proteome requires zinc for proper functioning [2]. Zinc acquisition from the environment, especially in zinc-limiting conditions, is mainly performed by the well-characterized ZIP family of plasma membrane transporters Zrt1p and Zrt2p [3], [4] and by the multi-metal transporter Fet4p [5]. Intracellular transport of zinc to organelles, a process associated with high-zinc growth conditions, is mediated by the cation diffusion facilitator families Zrc1p, Cot1p and Msc2p [5]–[8]. The expression of the majority of these transporters is directly regulated by the master zinc regulator Zap1p [9], a multi-zinc finger transcription factor [4] that specifically binds to zinc-responsive elements in the promoter regions of over 40 genes in the yeast genome, including *ZRT1* and *ZRT2* [10].

Zinc is an indispensable micronutrient for all organisms. Pathogenic microorganisms require zinc for successful growth and development of infectivity [11]. Therefore, mammalians limit zinc availability as a defensive strategy against invading pathogens by forming zinc complexes with proteins such as calprotectin [12], [13]. However, in comparison to the well-characterized microbial iron homeostasis system and its importance in host-pathogen interactions [14], [15], relatively little is known about the role of zinc in the host-pathogen interplay in the infection milieu. Zinc metabolism studies in pathogenic fungi have mainly focused on *Aspergillus fumigatus* and *Candida albicans*. The *A. fumigatus* transcription factor *ZafA* is a functional homolog of *S. cerevisiae* Zap1p, and its transcript levels are regulated by zinc availability. Moreover, *ZafA* is associated with the regulation of the zinc transporter-encoding genes *zrfA* and *zrfB*, further underscoring its role in zinc homeostasis. Mutant cells lacking *ZafA* display attenuated virulence, as assessed in murine models of aspergillosis [16]. The functional homologues of *S. cerevisiae* Zap1p in *C. albicans* were identified in two independent studies and were named Csr1p [17] and Zap1 [18], respectively. *C. albicans* mutant cells lacking Csr1p display a severe growth reduction in low-zinc environments and defects in filamentous growth, an important virulence-associated trait [17]. In addition, this protein positively regulates the expression of zinc transporters [18], further reinforcing its role in zinc homeostasis.

The basidiomycete yeasts *Cryptococcus neoformans* and *C. gattii* are the etiological agents of cryptococcosis, a life-threatening disease mostly characterized by meningoencephalitis. Cryptococcosis is a devastating disease in Africa and a major cause of death in immunosuppressed patients in many countries [19], [20]. *C. neoformans* var *grubii* (serotype A) is the most prevalent cause of human cryptococcosis, accounting for over 95% of cryptococcal cases worldwide, while *C. gattii* infections account for less than 1% of cryptococcosis cases [21]. However, *C. gattii* is usually associated with cryptococcosis in immunocompetent individuals [22]. In addition, cryptococcosis outbreaks caused by a hypervirulent strain of *C. gattii* on Vancouver Island [23] and in the USA [24], [25] reinforce the need for a thorough molecular characterization of the virulence determinants of this species. At least four well-characterized pathogenic determinants are shared by *C. neoformans* and *C. gattii*: (i) the presence of a polysaccharide capsule, (ii) the synthesis of a melanin-like pigment, (iii) the ability to proliferate at human body temperature and (iv) the ability to proliferate inside macrophages [26]. Nevertheless, only a few genes for other virulence determinants have been characterized in *C. gattii* to date [22].

Despite its importance, zinc metabolism is poorly characterized in both *C. neoformans* and *C. gattii*. This is in contrast to both iron metabolism [15] and copper metabolism [27], [28], which have been well-studied. Zinc chelation by calprotectin impairs the growth of *C. neoformans*, and this mechanism is also likely to be important during interactions between cryptococcal cells and neutrophils and other immune cells [29]. Here, we characterized the Z*AP1* gene and its role as a transcriptional regulator of zinc metabolism in *C. gattii*. *ZAP1* shares structural and functional features with other fungal zinc regulators, and functional analysis revealed that Zap1 regulates the expression of several genes involved in zinc metabolism. In addition, the results of this study demonstrate that Zap1 is necessary for key events in various cryptococcal pathogenesis-related mechanisms.

## Results

### Identification of the C_2_H_2_ Zn-finger transcription factor Zap1 in *C. gattii*

**Figure 1:**
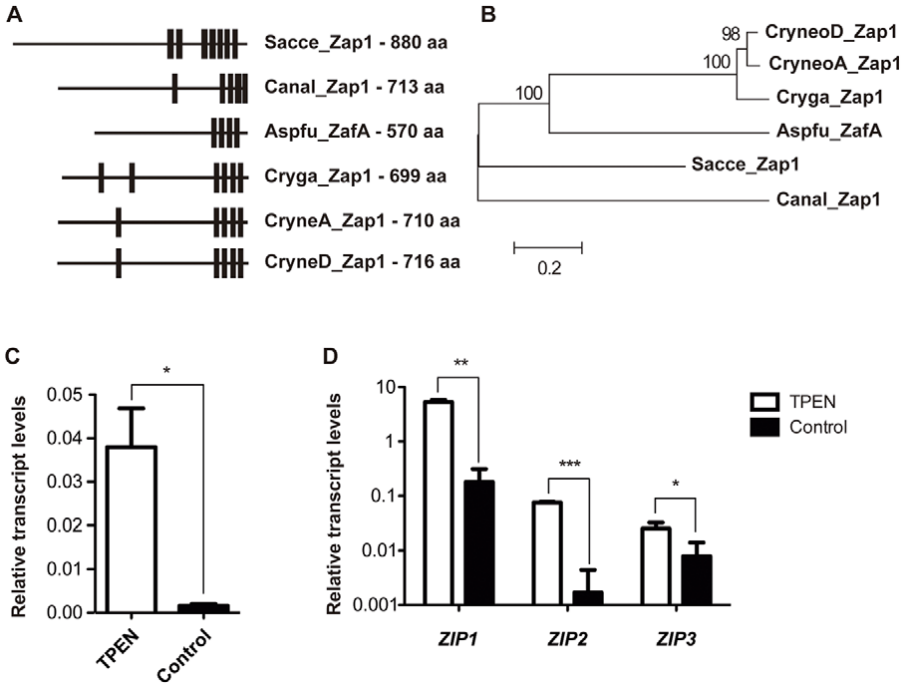
Identification of the *ZAP1* zinc regulator in *C. gattii.* A. The zinc finger domain architecture was evaluated using the ScanProsite tool employing the consensus sequence C-X(2,4)-C-X(12)-H-X(3,5)-H [16] in the *ZAP1* homologs of different fungi: *S. cerevisiae ZAP1* (Sacce_Zap1 – Genbank NP_012479.1), *A. fumigatus ZafA* (Aspfu_ZafA – Genbank ABJ98717.1), *C. albicans ZAP1* (Canal_Zap1 – Genbank XP_717199.1), *C. neoformans* serotype D *ZAP1* (CryneoD_Zap1 – Genbank XP_572252), *C. neoformans* serotype A *ZAP1* (CryneoA_Zap1 – Broad Institute CNAG_05392) and *C. gattii ZAP1* (Cryga_Zap1 – Broad Institute CNBG_4460). The zinc finger domain is represented by black bars, and the length of each protein sequence (in amino acids) is indicated to the right. B. Phylogenetic analysis applying the Neighbor-Joining method and including Zap1 sequences from distinct fungi. The bar marker indicates the genetic distance, which is proportional to the number of amino acid substitutions. C. Quantitative real time RT-PCR of *ZAP1* gene transcripts after growth of *C. gattii* in YNB with or without TPEN. D. Quantitative real time RT-PCR of *ZIP* gene transcripts after growth of *C. gattii* in YNB with or without TPEN. The measured quantity of the mRNA in each of these samples was normalized using the *Ct* values obtained for the actin gene. Data are shown as the mean ± SD from three experimental replicates of three biological replicates. ^*^ *P*<0.05. ^**^ *P*<0.01. ^***^ *P*<0.001.

Scrutiny of the *C. gattii* R265 genome [30] using the *S. cerevisiae* zinc finger metalloregulatory protein Zap1p revealed the presence of 25 predicted C_2_H_2_ zinc finger domain-containing proteins. Considering that *S. cerevisiae* Zap1p is characterized by a concentration of zinc fingers in its C-terminus [4], proteins without this feature were not considered for further analysis. The best-hit protein matching these criteria is encoded by the gene CNBG_4460, which contains four C_2_H_2_ domains. As previously demonstrated, *S. cerevisiae* Zap1p possesses seven zinc finger binding domains [4]. Thus, a direct comparison of the predicted CNBG_4460 encoded protein with the orthologs of the *C. neoformans* H99 and JEC21 strains was performed. The overall identity between proteins from the two *C. neoformans* strains was very high (>98%). However, when each predicted *C. neoformans* protein was compared to the putative *C. gattii* protein, the similarity was found to be only 78% (Figure S1), which may be due to a gap in the sequence corresponding to the C-terminus of the *C. gattii* protein. Analysis of the CNBG_4460 locus sequence relative to proteins from both *C. neoformans* strains was conducted employing the Gene Wise algorithm (http://www.ebi.ac.uk/Tools/Wise2/) to search for possible frameshift or annotation errors. The resulting sequence of the *C. gattii* Zap1 protein displayed increased identity with proteins from both *C. neoformans* strains (84%), and the similarity is likely due to the presence of an additional 70-bp exon (Figure S1). Moreover, RNA-seq confirmed the presence of an extra exon in the C-terminal coding region of the *C. gattii ZAP1* gene. In this way, the proposed annotation for the *C. gattii ZAP1* gene is quite identical to the *C. neoformans* orthologs (Figure S1). Thus, the *ZAP1* gene encodes a 699-aa protein containing six C_2_H_2_ zinc finger domains that are distributed along its sequence, as observed in fungal Zap1 functional homologs (Figure 1A). The domain architecture in *C. gattii* Zap1 protein is similar to those observed in *C. neoformans* functional homologs. Additionally, phylogenetic analysis (Figure 1B) including Zap1 sequences from different fungal species demonstrated that *C. gattii* Zap1 is highly similar to Zap1 from *C. neoformans* and *A. fumigatus* but less similar to Zap1 from *C. albicans*.

The expression of Zap1p is regulated by zinc levels in *S. cerevisiae* [4]. To further characterize the functional homology of *C. gattii* Zap1, the transcript levels from the *ZAP1* gene were assessed by qRT-PCR analysis using RNA isolated from *C. gattii* that were cultured in both the presence and the absence of the zinc chelator TPEN. A significant increase in *ZAP1* transcripts (25-fold) was detected in zinc-limiting conditions compared to control conditions (Figure 1C), suggesting a possible role for *C. gattii* Zap1 in zinc metabolism. An increase in the transcript levels of distinct ZIP family zinc transporters (*ZIP1* – CNBG_6066, *ZIP2* – CNBG_2209, and *ZIP3* – CNBG_5361) could also be detected in the same experimental conditions (Figure 1D). This result indicates the utility of TPEN as a tool to evaluate the zinc deprivation responsiveness of *C. gattii*, and confirms that *ZAP1* transcript levels can be regulated by zinc availability.

### *C. gattii* Zap1 regulates zinc transport and growth during zinc deprivation

**Figure 2:**
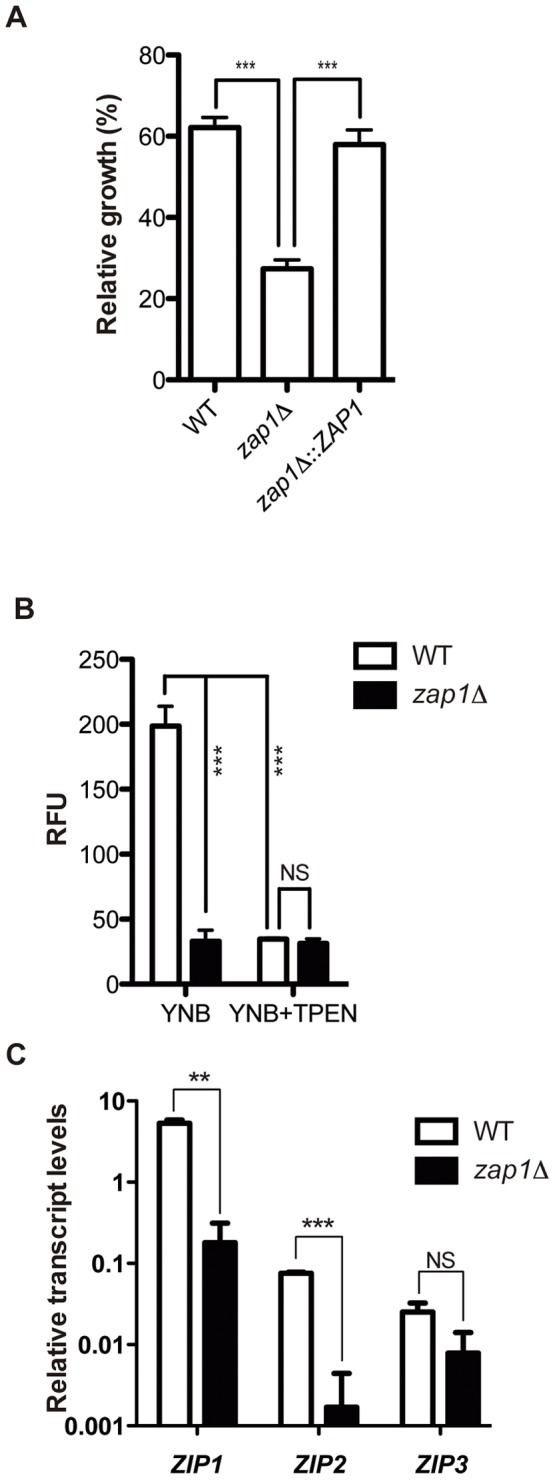
*C. gattii ZAP1* null mutants are defective in zinc metabolism. (A) Growth of the WT, *zap1*Δ mutant and *zap1*Δ::*ZAP1* complemented strains in low-zinc or control media was evaluated spectrophotometrically. The ratio between growth in low-zinc and control conditions is show as the mean ± SD from three biological replicates. (B) Fluorometric determination of intracellular zinc was accomplished using the probe Fluozin-1-AM. The relative zinc concentration was determined based on the fluorescence in WT or *zap1*Δ mutant cells cultured in YNB or YNB +10 μM TPEN as a control to determine the level of background fluorescence. Bars represent the mean of the cell count of normalized fluorescence levels. C. Quantitative real time RT-PCR of *ZIP* gene transcripts after growth of the *C. gattii* WT or *zap1*Δ mutant in YNB + TPEN. The measured quantity of the mRNA in each of the samples was normalized using the *Ct* values obtained for the actin gene. Data are shown as the mean ± SD from three experimental replicates of three biological replicates. ^**^ *P*<0.01. ^***^ *P*<0.001. NS, not significant.

To evaluate the function of Zap1 in *C. gattii*, null mutants and complemented strains were constructed. Knockout and complementation of the *ZAP1* gene were confirmed by both Southern blotting and RT-PCR analysis (Figure S2). To evaluate the role of *C. gattii* Zap1 in zinc homeostasis, the ability of WT, *zap1*Δ mutant and *zap1*Δ::*ZAP1* complemented strains to grow in zinc-limiting conditions (YNB containing TPEN) were assayed. Zinc deprivation induced decreased growth in all strains analyzed in comparison to their growth in zinc-rich medium. However, this growth reduction was much more pronounced in the *zap1*Δ mutant (Figure 2A). To evaluate the possibility that the growth arrest in *zap1*Δ mutants exposed to zinc deprivation conditions might be related to lower intracellular zinc levels, fluorometric analyses were performed with the cell permeable zinc probe Fluozin – 1 AM. Relative fluorescence levels of *zap1*Δ mutant were approximately 5 times lower than those of the WT strain and were comparable to background fluorescence levels obtained from the treatment of WT and *zap1*Δ mutant cells with the membrane permeable TPEN (Figure 2B). Because fungal Zap1 orthologs regulate the expression of ZIP family zinc transporters [16], [18], [31], qRT-PCR analyses were conducted to evaluate the relative transcript levels of three genes encoding ZIP domain containing proteins. The expression of two of these genes (*ZIP1* – CNBG_6066 and *ZIP2* – CNBG_2209) was regulated by Zap1 because the relative transcript levels of these genes were drastically reduced in the *zap1*Δ mutant cells compared to WT cells (Figure 2C). Collectively, these results confirm that *C. gattii* Zap1 plays a key role in the regulation of zinc homeostasis.

### *C. gattii* cells lacking Zap1 display alterations in oxidative stress pathways

**Figure 3:**
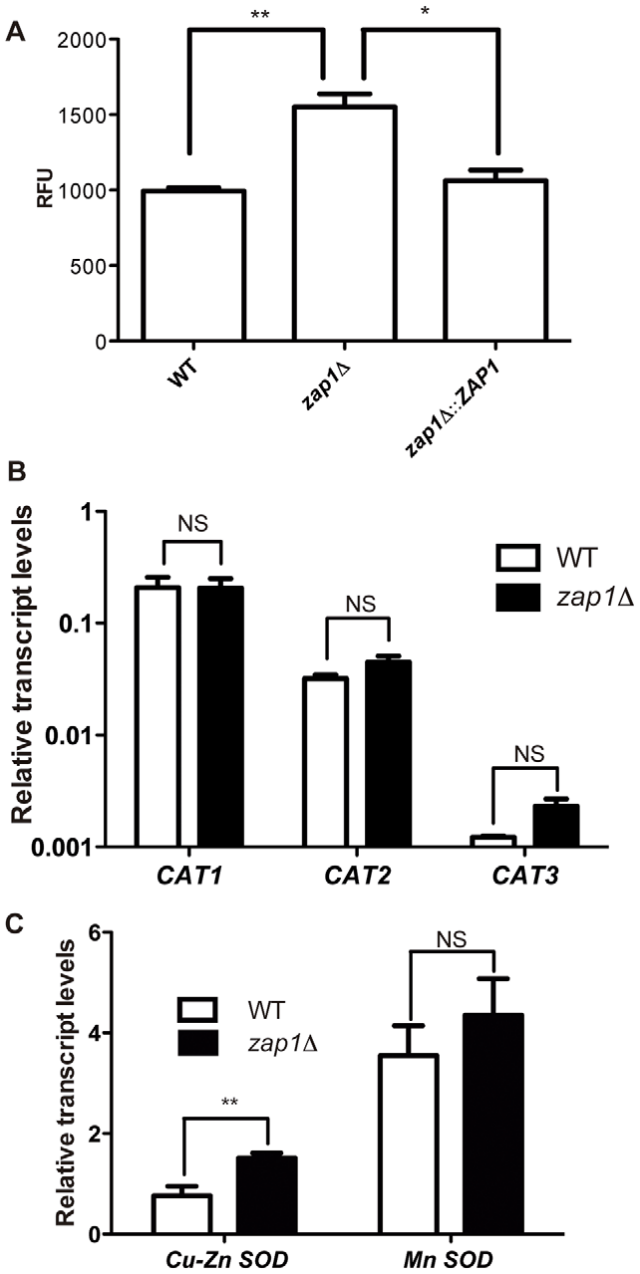
Disruption of *ZAP1* generates an imbalance in ROS metabolism. (A) Fluorometric determination of intracellular ROS levels employing the probe CM-H2DCFDA. The relative ROS levels were determined based on the fluorescence in WT, *zap1*Δ mutant or *zap1*Δ::*ZAP1* complemented cells cultured in YNB + TPEN. Bars represent the mean of the cell count with normalized fluorescence levels. (B) Quantitative real time RT-PCR of *CAT* gene transcripts after growth of *C. gattii* WT or *zap1*Δ mutant cells in YNB + TPEN. (C) Quantitative real time RT-PCR of *Mn-SOD* or *Cu/Zn-SOD* gene transcripts after growth of the *C. gattii* WT or *zap1*Δ mutant cells in YNB + TPEN. The measured quantity of the mRNA in each of the samples was normalized using the *Ct* values obtained for the actin gene. Data are shown as the mean ± SD from three experimental replicates of three biological replicates. ^*^ *P*<0.05. ^**^ *P*<0.01. NS, not significant.

Zinc deficiency generates a burst of oxidative stress in *S. cerevisiae* cells, and the adaptive responses to overcome some of the damage caused by reactive oxygen species (ROS) can be mediated by Zap1p [32]. Assays to evaluate the sensitivity of WT, *zap1*Δ mutant and *zap1*Δ::*ZAP1* complemented strains to hydrogen peroxide, menadione or T-BOOH revealed no differences in their relative growth when exposed to these distinct ROS generators (data not shown). However, assays employing the intracellular fluorescent ROS probe CM-H2CFDA revealed an accumulation of ROS in mutant cells, compared to the level in WT and complemented strains, after cultivation in YNB with TPEN (Figure 3A). To gain insight into this phenomenon, the relative transcript levels of three catalase- (*CAT1* – CNBG_4696, *CAT2*– CNBG_5786 and *CAT3* – CNBG_4667) and two superoxide dismutase (SOD)-encoding genes (*SOD1* – CNBG_0599 and *SOD2* – CNBG_2661) were measured by qRT-PCR. Comparison of the relative transcript levels of the *CAT* genes from WT and *zap1*Δ mutant cells revealed no statistically significant differences (Figure 3B). Nevertheless, a slight increase (1.9-fold) was observed in the levels of the Cu-Zn SOD encoded by CNBG_0599 but not in the Mn SOD encoded by CNBG_2661 (Figure 3C).

**Figure 4:**
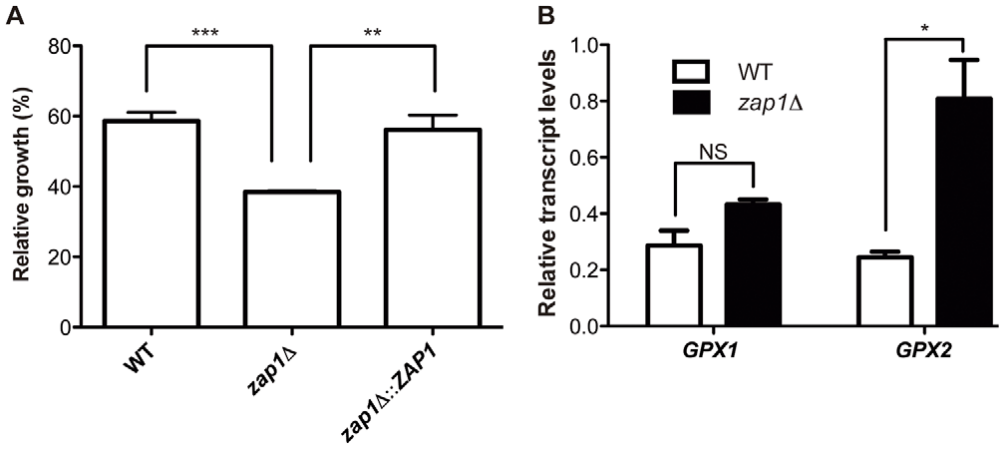
Lack of *ZAP1* leads to alterations in glutathione metabolism. (A) The WT, *zap1*Δ mutant and *zap1*Δ::*ZAP1* complemented strains were incubated in YNB or YNB +0.5 mM DEM. After 24 h of incubation, the cell density was spectrophotometrically determined. The ratio between growth in DEM and control conditions is shown as the mean ± SD from three biological replicates. (B) Quantitative real time RT-PCR of *GPX* gene transcripts after growth of *C. gattii* WT or *zap1*Δ mutant cells in YNB + TPEN. The measured quantity of the mRNA in each of the samples was normalized using the *Ct* values obtained for the actin gene. Data are shown as the mean ± SD from three experimental replicates of three biological replicates. ^*^ *P*<0.05. ^**^ *P*<0.01. ^***^ *P*<0.001. NS, not significant.

**Figure 5:**
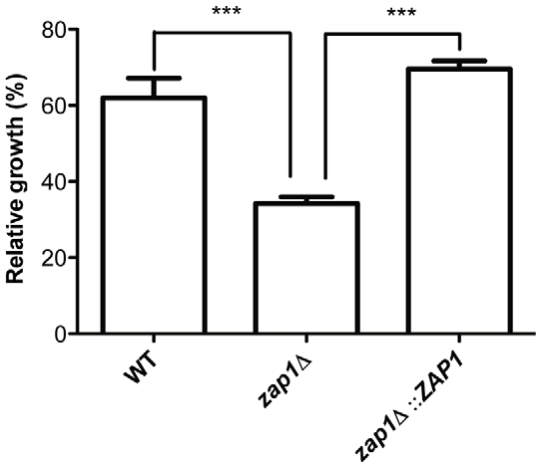
*C. gattii ZAP1* gene null mutant shows defects in the response to RNS. The WT, *zap1*Δ mutant and *zap1*Δ::*ZAP1* complemented strains were incubated in YNB or YNB +1 mM DETA-NONOate. After 24 h of incubation, the cell density was spectrophotometrically determined. The ratio between growth in DEM and control conditions is shown as the mean ± SD from three biological replicates. ^***^ *P*<0.001.

The accumulation of intracellular ROS in *C. gattii zap1*Δ mutant cells suggested that other mechanisms might also be involved in the regulation of ROS homeostasis in such cells. The *S. cerevisiae* Zap1p is also known for its role in the regulation of the expression of distinct genes involved in sulfur and glutathione metabolism [33], [34]. To test this possibility, the sensitivity of *zap1*Δ mutant cells to the glutathione depletion agent diethyl malate (DEM) was examined. While the addition of DEM to WT or *zap1*Δ::*ZAP1* complemented strains reduces their growth to approximately 60% of the level of non-treated cultures, it caused a more intense growth inhibition in the *zap1*Δ mutant strain to approximately 40% of the level of non-treated cultures (Figure 4A). This result suggests that the lack of *ZAP1* leads to a reduction in glutathione levels in these cells. In an attempt to correlate the proposed diminished glutathione levels with the elevated intracellular ROS levels, the relative transcript levels of two glutathione peroxidase (GPx)-encoding genes (*GPX1* – CNBG4202 and *GPX2* – CNBG5153) were evaluated. GPxs catalyze the reduction of hydrogen peroxide with the consumption of reduced glutathione, yielding oxidized glutathione [35]. The relative *GPX2* gene transcript levels were 4 times higher in the *zap1*Δ mutant compared to WT cells (Figure 4B), confirming an imbalance in glutathione and ROS metabolism in the absence of *ZAP1*. In addition, analysis of the sensitivity of WT cells, *zap1*Δ mutants and *zap1*Δ::*ZAP1* complemented strains to the reactive nitrogen species (RNS) generator DETA-NONOate revealed that the absence of *ZAP1* gene activity leads to a marked decrease in the viability of the *zap1*Δ mutant compared to the WT or complemented strains (Figure 5).

### Effects of *ZAP1* deletion on *C. gattii* virulence

**Figure 6:**
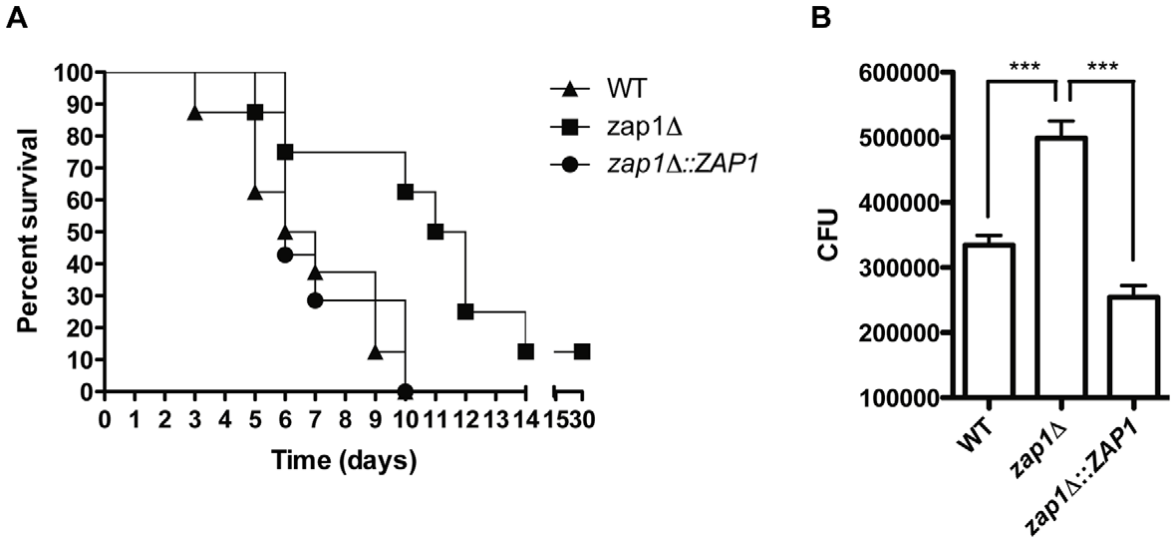
Zap1 is required for full *C. gattii* virulence in mice and influences phagocytosis by macrophages. (A) Virulence assay of WT, *zap1*Δ mutant and *zap1*Δ::*ZAP1* complemented strains in an intranasal inhalation infection model with BALB/c mice. (B) CFU counts after macrophage infection with WT, *zap1*Δ mutant and *zap1*Δ::*ZAP1* complemented strains. ^***^ *P*<0.001.

The *zap1*Δ strain was evaluated for its ability to synthesize melanin and capsule and for its ability to grow at 37°C, as these are the most well studied virulence factors of *C. gattii* [26]. The lack of *ZAP1* does not interfere with any of these traits (Figure S3), but a decrease in the ability to cause experimental cryptococcosis was observed in *zap1*Δ cells. Using an intranasal model of murine infection, we found that mice infected with the *ZAP1* null mutant survived longer (median survival 11.5 days) than those infected with the WT (P = 0.0078) and complemented strains (P = 0.0253) (median survival 6 and 6.5 days, respectively; Figure 6A). In this context, interactions between yeast cells and macrophages play a pivotal role in the pathobiology of *C. neoformans* [36]. To evaluate whether the lower virulence of the *zap1*Δ mutant is associated with changes in the interplay between macrophages and *C. gattii*, phagocytosis assays were performed employing the macrophage cell-like RAW264.7 line. Assays with the WT, *zap1*Δ mutant and *zap1*Δ::*ZAP1* complemented strains revealed that cells lacking *ZAP1* have increased CFU counts after 18h of interaction with macrophages (Figure 6B). In conjunction, these results confirm the role of *ZAP1* in *C. gattii* virulence.

### *ZAP1* regulates the expression of zinc transporters, oxidative stress-related proteins and zinc-binding proteins

To evaluate the response of the *ZAP1* regulon to zinc deprivation, RNA-seq analyses were conducted using RNA isolated from WT or *zap1*Δ mutant cells after a 2-h exposure to TPEN, as this condition promotes the intense expression of *ZAP1* and *ZIP* zinc transporters (Figure 1C and 1D). Analysis of the two transcriptomes revealed a total of 183 and 328 genes that were significantly (p<0.05) up-regulated and down-regulated, respectively, in WT cells compared to *zap1*Δ mutant cells. As expected, two ZIP zinc transporters were found among the up-regulated genes, providing a positive control for the analysis. In addition, one gene related to nitrosative stress, the flavohemoglobin encoding gene, was also up-regulated in WT cells (Table 1). The full list of *ZAP1*-regulated genes and the corresponding fold-changes are shown in Table S1. To further characterize the genes regulated by *ZAP1* in *C. gattii*, a screen for genes encoding putative zinc-binding proteins was conducted, employing gene ontology (GO) classification after analysis on the UFO server [37]. Among the 183 WT up-regulated genes analyzed, four zinc-binding proteins were detected, and all are transcription factors containing distinct zinc finger domains (Table 1). However, when the 328 WT down-regulated genes were subjected to the same analysis, thirteen different genes encoding putative zinc-binding proteins were identified. The majority of these genes encode proteins with dehydrogenase activity, and only two encode zinc finger transcription factors (Table 1).

**Table 1:** List of selected *ZAP1*-regulated genes in *C. gattii*.

Category/ Accession^A^	Description^A^	PFAM^A^	Fold Change^B^
**Transport**			
CNBG_2209	zrt1 protein	ZIP zinc/iron transport family	6.96
CNBG_6066	zinc ion transporter	ZIP zinc/iron transport family	6.42
CNBG_4611	sodium: inorganic phosphate symporter	Phosphate transporter family	2.05
**Oxidative metabolism**			
CNBG_3964	flavohemoglobin	Oxidoreductase NAD-binding domain	2.24
**Sulfate metabolism**			
CNBG_1788	glutathione S-transferase	Mak16 protein	2.08
CNBG_6075	S-adenosylhomocysteine hydrolase	S-adenosyl-L-homocysteine hydrolase	2.00
CNBG_6043	glutathione transferase	N/A	−1.87
CNBG_1291	cystathionine beta-synthase	Pyridoxal-phosphate dependent enzyme	−4.34
**Zinc-binding^C^**			
CNBG_1642	transcription factor IIIb	TFIIB zinc-binding; Brf1-like TBP-binding domain	3.67
CNBG_2093	RNA polymerase III smallest subunit	Transcription factor S-II (TFIIS)	1.87
CNBG_3335	DNA-directed RNA polymerase I polypeptide	Transcription factor S-II (TFIIS)	2.10
CNBG_0427	conserved hypothetical protein	Zinc-binding dehydrogenase	−2.21
CNBG_1120	conserved hypothetical protein	Putative GTPase activating protein for Arf	−2.00
CNBG_1321	cytoplasm protein	Zinc-binding dehydrogenase	−0.62
CNBG_2992	alcohol dehydrogenase	Alcohol dehydrogenase GroES-like domain	−0.81
CNBG_3576	quinone oxidoreductase	Alcohol dehydrogenase GroES-like domain	−2.00
CNBG_3878	zinc-binding dehydrogenase	Alcohol dehydrogenase GroES-like domain	−2.73
CNBG_3919	xylitol dehydrogenase	Alcohol dehydrogenase GroES-like domain	−2.48
CNBG_4844	conserved hypothetical protein	Fungal Zn(2)-Cys(6) binuclear cluster domain	−1.59
CNBG_4875	R,R-butanediol dehydrogenase	Alcohol dehydrogenase GroES-like domain;	−2.67
CNBG_5308	conserved hypothetical protein	Zinc finger, ZZ type	−1.81
CNBG_5559	conserved hypothetical protein	LIM domain	−1.80
CNBG_6001	extracellular elastinolytic metalloproteinase	Fungalysin metallopeptidase (M36);	−2.70
CNBG_6010	mannose-6-phosphate isomerase	Phosphomannose isomerase type I	−2.41

A: Gene accessions, descriptions and PFAM descriptions were obtained from the Broad Institute Database.

B: Data are presented as the ratio of FPKM (fragments per kilobase of exon per million fragments mapped) of genes in WT cells compared to their expression in *ZAP1* mutant cells during growth in YNB + TPEN using a log scale.

C: Zinc-binding proteins were selected based on UFO analysis [37] using the predicted protein sequences of significantly differentially expressed genes in the WT or *zap1*Δ strains as input. The sequences that contained the GO term “zinc ion binding” (GO:0008270) were selected for further functional classification.

## Discussion

Zinc is a fundamental micronutrient in cell physiology, as it is a key component of the cores of several proteins [38]. Fungal cells have evolved a regulatory mechanism to acquire and distribute zinc inside cells [32]. In all fungal systems characterized to date, including *S. cerevisiae, C. albicans* and *A. fumigatus*, the Zap1p transcription factor and its functional homologs control zinc homeostasis through the modulation of zinc transporter and zinc-binding protein expression [4], [16]–[18]. In the present study, the functional homolog of *S. cerevisiae* Zap1p was characterized in *C. gattii*, allowing us to analyze the influence of zinc in cryptococcal virulence. Four lines of evidence support the assumption that Zap1 is a zinc-responsive transcriptional regulator that is responsible for zinc homeostasis in *C. gattii*. First, *ZAP1* transcript levels increase in response to zinc deprivation. Second, *in silico* analysis of the predicted Zap1 protein sequence identified several C_2_H_2_ zinc finger domains that are common to all fungal Zap1 functional homologs described to date [4], [16], [17]. Third, Zap1 is associated with the regulation of zinc transporter expression because in *C. gattii ZAP1* null mutants, the relative transcript levels of genes encoding the ZIP family transporters are drastically reduced compared to WT cells. Fourth, the growth of the *C. gattii* zap1Δ strain is reduced in zinc-limiting conditions. Additionally, *C. gattii* null mutants for the *ZAP1* gene display attenuated virulence in the intranasal murine model of cryptococcosis, and these mutants associate more with macrophages than do WT and complemented strains in phagocytosis assays. The modulation of virulence was also demonstrated for mutants of the *ZAP1* homolog in *A. fumigatus* (ZafA gene) [16], further indicating the importance of zinc homeostasis in the virulence of fungal pathogens.

At least three transcription factors that regulate metal homeostasis were described in the phylogenetically related yeast *C. neoformans*. The *CIR1* and *HAPx* genes are involved in the regulation of iron homeostasis [39], [40], and *CUF1* regulates copper metabolism in *C. neoformans* [28]. Lack of *CIR1* in *C. neoformans* yields cells with enhanced melanization, decreased capsule size, and temperature-sensitive growth [40], while *C. neoformans CUF1* null mutants are hypomelanized [28]. The *CUF1* and *CIR1* mutant phenotypes contribute to their reduced virulence, as assayed by murine models of cryptococossis [28], [40]. The analyses presented here demonstrate that *C. gattii ZAP1* null mutants also display reduced virulence in the same animal models and that this phenotype could not be associated with any detectable alteration in the classical virulence traits, such as melanin and capsule formation and host temperature growth. The results observed with *C. gattii ZAP1* are in agreement with those described for *C. neoformans*, in which the lack of *ZAP1* leads to a severe defect in murine infectivity and reduced melanization [41]. A recent report evaluated the phenotypes associated with the knockout of distinct genes encoding functional homologs belonging to distinct functional classed in both *C. neoformans* and *C. gattii*. Despite conservation in phenotypes concerning the predicted activity of the encoded protein, subtle differences could be found when compared to the classical virulence traits among the species [22]. It is therefore reasonable to assume that the decrease in virulence of *C. gattii ZAP1* mutants can be attributed to defects in their ability to grow in low-zinc conditions and to take up zinc from the environment. Indeed, the reduction in zinc availability in the infection milieu is a host strategy designed to hamper pathogen replication [11]. For instance, a decrease in cytoplasmic zinc concentration is observed in murine macrophages infected with *Histoplasma capsulatum* or treated with cytokines that induce antimicrobial activity [42]. It has also been reported that abscesses resulting from infection with the bacterium *Staphylococcus aureus* are rich in the SB100 zinc-binding protein calprotectin, leading to zinc chelation and thereby reducing zinc availability to the pathogen [12]. Furthermore, the exposure of *C. neoformans* cells to calprotectin leads to growth inhibition and cell death [29]. Altogether, these data reinforce the dependence of *C. neoformans* and *C. gattii* on zinc availability for development within the host milieu.

We observed that *C. gattii ZAP1* mutant cells display several defects in their ability to handle oxidative stress. In addition to the accumulation of intracellular ROS, such cells display alterations in glutathione metabolism. For instance, *C. gattii ZAP1* mutant cells display increased *GPX2* transcript levels and are more sensitive to diethyl malate compared to WT cells. This suggests a decreased concentration of intracellular glutathione in *C. gattii ZAP1* mutant cells. In light of these results, we hypothesize that as an adaptive response to the zinc deprivation-induced accumulation of intracellular ROS levels, *C. gattii ZAP1* mutant cells modulate the ROS balance through glutathione metabolism. Via the activity of GPx, cells can detoxify ROS with the concomitant consumption of glutathione [35]. Accordingly, *GPX* null mutants of *C. neoformans* are hypersensitive to oxidative stress [43]. It is well documented that zinc depletion results in enhanced ROS levels inside *S. cerevisiae* cells [32], [44]. As an adaptive response to elevated ROS levels caused by zinc deprivation, *S. cerevisiae* cells activate the expression of the *TSA1* gene, which encodes a peroxiredoxin, to degrade hydroperoxides [44]. The *ZAP1* gene from *S. cerevisiae* is also involved in the regulation of the catalase encoding gene *CTT1* in low-zinc conditions, suggesting that this enzyme plays a role in ROS detoxification under conditions of zinc deprivation [31]. The uptake and metabolism of sulfate is largely dependent upon *ZAP1* activity in *S. cerevisiae* and is repressed in zinc-limiting conditions [33]. Methionine, cysteine, and (most likely) other metabolites from sulfur metabolism, including glutathione, are found at lower concentrations in zinc-limited *S. cerevisiae* cells, suggesting that the detoxification of ROS in zinc-limiting conditions also relies on proper glutathione metabolism [32]. Altogether, these findings suggest that *C. gattii* likely evolved a Zap1-independent strategy to cope with the elevated ROS levels caused by zinc-limiting conditions, unlike those observed in *S. cerevisiae*.

The generation of RNS is a common strategy used by immune cells to hamper the development of *C. neoformans* and other fungi [45]. The successful growth and virulence of *C. neoformans* in nitrosative conditions, both *in vitro* and *in vivo*, depends on the activity of flavohemoglobin denitrosylase and S-nitrosoglutathione reductase, which are encoded by the *FHB1* and *GNO1* genes, respectively [46], [47]. *C. gattii ZAP1* null mutants are more sensitive to the RNS generator DETA-NONOate, possibly as a consequence of reduced transcript levels of the *FBH1* ortholog in *C. gattii*, as observed in our transcriptome analysis. Therefore, in addition to defects in zinc transport and metabolism, the attenuated virulence observed in the *C. gattii ZAP1* null mutants may also be associated with defects in dealing with nitrosative stress.

The comparison of the *C. gattii ZAP1* regulon with its counterparts from *S. cerevisiae* and *C. albicans* revealed some overlapping circuits. The ZIP family of zinc transporters is positively regulated by *ZAP1* in yeasts [18], [31]. However, Zap1 regulates several other genes that are not necessarily associated with zinc homeostasis. When specifically analyzing the zinc-binding proteins, as inferred from their GO annotations, we observed that Zap1 is a negative regulator of several zinc-binding proteins. The results presented here show that some alcohol dehydrogenases are downregulated in WT cells compared to the *ZAP1* null mutants. The same expression pattern is also observed in *S. cerevisiae* and *C. albicans* [18], [31]. This strategy could represent an adaptation to low zinc availability named “zinc conservation” [32], in which other metalloproteins necessary to survive in such conditions are preferentially associated with zinc. Alcohol dehydrogenases are among the most abundant zinc-binding proteins in the cell, representing a significant proportion of the total cellular zinc [32]. As a result, the reduced expression of some zinc-binding proteins would make zinc available for other proteins, such as Cu/Zn SODs, as a strategy to cope with the harsh conditions of a zinc-limiting milieu.

In conclusion, this report describes the identification and characterization of Zap1, a general regulator of zinc homeostasis in *C. gattii*. Two key events are responsible for the observed reduced virulence of *C. gattii ZAP1* null mutants in the intranasal murine model of infection: the reduced zinc load in cells and the corresponding increase in intracellular ROS. The *C. gattii* Zap1 regulon includes zinc transporters and several zinc-binding proteins, and there is some conservation between it and the regulatory circuits of other yeast Zap1 regulons.

## Materials and Methods

### Ethics statement

The use of animals in this work were performed with approval of The Universidade Federal do Rio Grande do Sul Ethics Committee for Use of Animals (CEUA – protocol number 19801). Mice were housed in groups of four in kept in filtered top ventilated cages, maintained on 12 h dark/light cycle, with food and water *ad libitum*. The animals were cared according to the Brazilian National Council for Animal Experimentation Control (CONSEA) and Brazilian College of Animal Experimentation (COBEA) guidelines. All efforts to minimize animal suffering were made. Before mortality analysis, mice were intraperitoneally anesthetized with 100 mg/kg Ketamine and 16 mg/kg Xylazine. Mice were analyzed twice daily for any signals of suffering, defined by weight loss, weakness or inability to obtain feed or water. In the first signals of suffering, mice were humanely sacrificed.

### Strains and culture conditions

The *C. gattii* strain R265 was used in this work. It was routinely cultured in YPD (2% glucose, 2% peptone, and 1% yeast extract). Agar was added to a final concentration of 1.5% when solid media was used. YPD plates supplemented with 100 µg/ml nourseothricin were used to select for the *C. gattii zap1*Δ mutant strain. YPD +200 µg/ml hygromycin was used to select for the *C. gattii zap1*Δ::ZAP1 complemented strain. Phenotypic assays were conducted in Yeast Nitrogen Base (YNB, without amino acids and ammonium sulfate) with the addition of asparagine to a final concentration of 40 mM. The murine macrophage-like cell line was used for the evaluation of phagocytosis-related phenotypes. It was routinely cultured in Dulbecco's Modified Eagle Medium (DMEM) with 10% heat-inactivated fetal bovine serum (FBS) in a humidified incubator at 37°C and 5% CO_2_.

### *In silico* identification and characterization of *C. gattii ZAP1*

The putative *C. gattii ZAP1* gene was identified by BLAST analysis of the Broad Institute *C. gattii* R265 database using the *S. cerevisiae* Zap1p sequence as a query (NCBI accession number NP_012479.1). Amino acid sequences of Zap1 orthologs from *S. cerevisiae*, *C. albicans*, *A. fumigatus*, *C*. *gattii* and *C. neoformans* were aligned using ClustalX2 [48]. Mega5 was utilized for phylogenetic analysis applying the Neighbor-Joining method; tree architecture was inferred using 1000 bootstraps [49]. Searches for zinc-binding domains were conducted using the ScanProsite tool [50].

### Construction of knockout and complemented *C. gattii ZAP1* strains

The Delsgate methodology was used to construct the *ZAP1* gene inactivation allele by employing the vector pDONR-NAT, as previously described for *C. neoformans* [51], [52]. The nourseothricin resistance cassette from pAI4 was subcloned into the *Eco*RV site of pDONR201 (Gateway donor vector, Invitrogen) to construct plasmid pDONR-NAT. The 5′ and 3′ *ZAP1* flanking sequences (953 and 801 bp, respectively) were PCR-amplified and gel purified (Illustra GFX PCR DNA and Gel Band Purification kit, GE Healthcare). The plasmid pDONR-NAT and each PCR product were mixed at equimolar ratios in a BP clonase reaction according to manufacturer's instructions (Invitrogen). This reaction was then transformed into *Escherichia coli* OmniMAX 2-T1 cells. The inactivation construct plasmid was linearized by I-*Sce*I digestion prior to *C. gattii* biolistic transformation [53]. For complementation, a 5-kb fragment spanning the *ZAP1* gene was amplified from *C. gattii* R265 DNA and subcloned into the *Eco*RV site of pJAF15, and the plasmid was transformed into the *C. gattii zap1*Δ strain. Correct integration of the inactivation cassette into the WT *ZAP1* locus was evaluated by Southern blot and RT-PCR analysis. The primers used in these constructions are listed in Table S2.

### Phenotypic assays

The viability of mutant cells in zinc-limiting media was assessed by pre-growing the WT, *zap1*Δ and *zap1*Δ::ZAP1 strains in YPD overnight (30°C). The cells were then washed with water and 1×10^6^ cells were inoculated in YNB containing 10 μM TPEN or YNB containing 10 μM TPEN and 10 μM ZnCl_2_. After 24 h of incubation at 30°C, the OD_600_ was determined. To evaluate the viability of cells when exposed to other chemicals, the cells were washed after the YPD incubation, and 1×10^6^ cells were inoculated in YNB with various concentrations of diethyl malate or DETA-NONOate. After 24 h of incubation at 30°C, the OD_600_ was determined. Capsule formation was examined by microscopy after incubation for 24 h (37°C and 5 % CO_2_) in DMEM media with 10 % FBS that was prepared with India ink. Relative capsule sizes were defined as the ratio between the capsule thickness and cell diameter. ImageJ software was utilized to determine the capsule measurements of 100 cells of each strain. Niger seed medium plates were used for melanin synthesis evaluation.

### RNA-seq analysis

WT and *zap1*Δ cells were pre-grown in YPD media (30°C for 24 h) and then inoculated in YNB media for a further period of growth (30°C for 18 h). YNB + 10 μM TPEN was inoculated with 1×10^7^ cells/mL and incubated for additional 4 h at 30°C. Cells were collected by centrifugation (10,000 g for 10 min), and RNA was isolated by Trizol (Invitrogen) after cellular lysis via mortar and pestle. RNA integrity and concentration were assessed by electrophoresis on a 1% agarose gel and by fluorometry analysis using a Qubit fluorometer and a Quant-iT RNA assay kit according to the manufacturer's instructions (Invitrogen). mRNA was purified from total RNA samples, processed and sequenced using Solexa technology on an Illumina Genome Analyzer GAII (Fasteris Life Sciences SA, Plan-les-Ouates, Switzerland). The resulting fastq files were aligned to the *C. gattii* R265 reference sequence [30] with the help of Tophat [54]. Aligned transcripts were quantified using cufflinks [55] with the current annotation of the *C. gattii* R265 genome provided by the Broad Institute. Differential expression was evaluated by the cuddiff module of cufflinks with a False Discovery Rate (FDR) set at 5%. Genes with an FDR corrected p-value <0.05 were considered to be statistically significant. Functional classification against PFAM and GO of differentially expressed genes was performed using the web server UFO [37].

### Real time RT-PCR analysis

RNA samples were prepared as above. cDNAs were prepared from DNAse (Promega)-treated total RNA samples (500 ng) with ImProm-II Reverse transcriptase (Promega) using oligo-dT. qRT-PCR was performed on a Real-time PCR StepOne Real-Time PCR System (Applied Biosystems) with thermal cycling conditions set with an initial step at 95°C for 5 min followed by 40 cycles at 95°C for 15 s, 55°C for 15 s and 60°C for 60 s. Platinum SYBR green qPCR Supermix (Invitrogen) was used as a reaction mix, supplemented with 5 pmol of each primer and 1 μl of the cDNA template in a final volume of 25 μl. All experiments were performed using three independent cultures, and each cDNA sample was analyzed in triplicate with each primer pair. Melting curve analysis was performed at the end of the reaction to confirm the presence of a single PCR product. Data were normalized to actin cDNAs amplified in each set of PCR experiments. Relative expression was determined by the 2^−*ΔCT*^ method [56]. Statistical analyses were conducted via a two-tailed Student's t-test. The primers used in these analyses are listed in Table S2.

### ROS measurement

Cells were grown overnight in YPD medium at 30°C, washed in YNB and incubated for an additional 24 h in YNB. Cells were washed and inoculated at a cell density of 1×10^7^ cells/mL in fresh YNB +10 μM TPEN. The acetoxymethyl ester of dichlorodihydrofluorescein diacetate (CM-H2DCFDA) (Molecular Probes) was added to a final concentration of 10 µM and incubated for an additional 2 h to load the dye into cells. After 2 h, the cells were washed with PBS and analyzed for fluorescence determination using a SpectraMax M4 plate reader fluorometer (Molecular Devices) with the emission and excitation wavelengths set at 488 and 520 nm, respectively. Fluorescence values were normalized to cell count, based on the OD_600_ determination. Statistical analyses were conducted with a two-tailed Student's t-test.

### Intracellular zinc level determinations

Cells were cultivated for ROS assessment. After a 24-h incubation in YNB, cells were washed and inoculated at a density of 1×10^7^ cells/mL in fresh YNB. The acetoxymethyl ester of Fluozin-1 (Fluozin-1 AM – Molecular Probes) was added to a final concentration of 10 µM and incubated for an additional 2 h to load the dye into the cells. A control experiment was conducted in which TPEN was included to evaluate the fluorescence background. After 2 h, the cells were washed with PBS and analyzed for fluorescence determination in a SpectraMax M4 plate reader fluorometer (Molecular Devices) with its emission and excitation wavelengths set to 495 and 517 nm, respectively. Fluorescence values were normalized to the cell count, as determine by OD_600_ measurement. Statistical analyses were conducted using a two-tailed Student's t-test.

### Macrophage assays

Phagocytosis assays were conducted to evaluate the susceptibility of WT, *zap1*Δ and *zap1*Δ::ZAP1 cells to the antifungal action of phagocytes. Macrophage-like RAW264.7 cells were seeded at a density of 1×10^5^ cells/100 μl of DMEM supplemented with 10% FBS in each well of the 96-well culture plates (TPP). After 24 h of incubation (37°C and 5% CO_2_), the medium was replaced with fresh medium containing 1×10^6^ cells of each fungal strain, obtained after a 24-h incubation in YPD and extensive washing in PBS. The plates were further incubated (18 h, 37°C, and 5% CO_2_), and yeast cells that were not associated with the macrophages were removed by PBS washes. Fungal survival was evaluated after macrophage lysis with sterile ice-cold Milli-Q water and subsequent plating on YPD for CFU determination. This assay was performed in triplicate for each strain. A Student's t-test was used to determine the statistical significance of the observed differences in fungal survival.

### Survival assays

Virulence studies were conducted as previously described [51]. Briefly, fungal cells were cultured in 50 ml YPD medium at 30°C overnight with shaking, washed twice and re-suspended in PBS. Groups of eight female BALB/c mice (approximately 5 weeks old) intraperitoneally anesthetized with 100 mg/kg Ketamine and 16 mg/kg Xylazine were infected with 1×10^7^ yeast cells suspended in 50 μl PBS and monitored twice daily for moribund signals. We used a high *C. gattii* inoculum since BALB/c mouse is moderately resistant to cryptococcal infection [57]. The median survival values were calculated by Kaplan–Meier survival analysis. Animal studies were approved by the Federal University of Rio Grande do Sul Ethics Committee.

## Supporting Information

Figure S1 ***In silico*** **characterization of** ***C. gattii*** **Zap1.** Multiple sequence alignment of the Zap1 ortholog sequences of *C. neoformans* serotype D Zap1 (CryneoD_Zap1 – Genbank XP_572252) and *C. neoformans* serotype A Zap1 (CryneoA_Zap1 – Broad Institute CNAG_05392) with the *C. gattii* Zap1 Broad Institute (A) and the proposed sequences (B). The predicted zinc fingers are shown as shaded black boxes. (C) Comparison of the CNBG_4460 locus (Supercontig 11: 588643–591042) automatic annotation and the proposed annotation with the annotated sequences of *ZAP1* from *C. neoformans* serotype D (CryneoD_*ZAP1* – Genbank NC_006693) and *C. neoformans* serotype A (CryneoA_*ZAP1* – Broad Institute CNAG_05392). Exons are depicted with arrows and arrowheads.
(TIF)

Figure S2 **Construction of the** ***C. gattii ZAP1*** **gene knockout and complemented strains.** A. *ZAP1* deletion scheme. TV represents the targeting vector constructed by Delsgate methodology. 5 ZAP1 and 3 ZAP1 represent the 5′ and 3′ flanking regions of the *ZAP1* gene, respectively. 5F and 5R: primers utilized to amplify the 5′ flanking region of *ZAP1*. 3F and 3R: primers utilized to amplify the 3′ flanking region of *ZAP1*. Nat: cassette that confers nourseothricin resistance. WT represents the wild type locus of the *ZAP1* gene in the R265 strain. *Δ* represents the *ZAP1* locus in the *zap1* mutant strain. The cleavage sites of the *Bgl*II restriction enzyme are indicated. B. Confirmation by Southern blotting. Genomic DNA (10 μg) from WT (lane 1), *zap1*Δ::*ZAP1* complemented (lane 2) and *zap1*Δ mutant (lane 3) strains was digested with *Bgl*II. The 3′ flanking region was used as the probe for Southern hybridization. Numbers on the left indicate the hybridization signal sizes based upon the position of the molecular size marker. C. Semi-quantitative RT-PCR using cDNA from WT, *zap1*Δ mutant and *zap1*Δ::ZAP1 complemented strains as the template. RNA samples were used as templates for reactions employing (+) reverse transcriptase. Control reactions without reverse transcriptase addition (−) were used to confirm the absence of genomic DNA. The upper panel shows the *ZAP1* amplicons, while the lower panel shows the *ACT1* amplicons (loading control).
(TIF)

Figure S3 **Analysis of virulence-related phenotypes of the** ***C. gattii ZAP1*** **null mutant.** (A) Melanin production was assessed by plating ten-fold serial dilutions of WT, *zap1*Δ mutant and *zap1*Δ::*ZAP1* complemented strains in niger seed agar and incubating for 48 h. (B) Ability to replicate at body temperature was assessed by plating ten-fold serial dilutions of WT, *zap1*Δ mutant and *zap1*Δ::*ZAP1* complemented strains in YNB agar and incubating at 30 or 37°C for 24 h. (C) Capsule production was evaluated by analysis of the capsule/cell ratio of 100 distinct cells from WT, *zap1*Δ mutant and *zap1*Δ::*ZAP1* cells cultured in capsule-inducing conditions.
(TIF)

Table S1 **Complete list of Zap1-regulated genes with at least 2-fold differential expression.**
(XLSX)

Table S2 **List of primers used in this work.**
(DOCX)
