# Supplementary figures and images for: Zap1 Regulates Zinc Homeostasis and Modulates Virulence in Cryptococcus gattii
Source: PLoS One. 2012 Aug 20;7(8):e43773. doi: 10.1371/journal.pone.0043773 (PMC3423376; doi:10.1371/journal.pone.0043773)

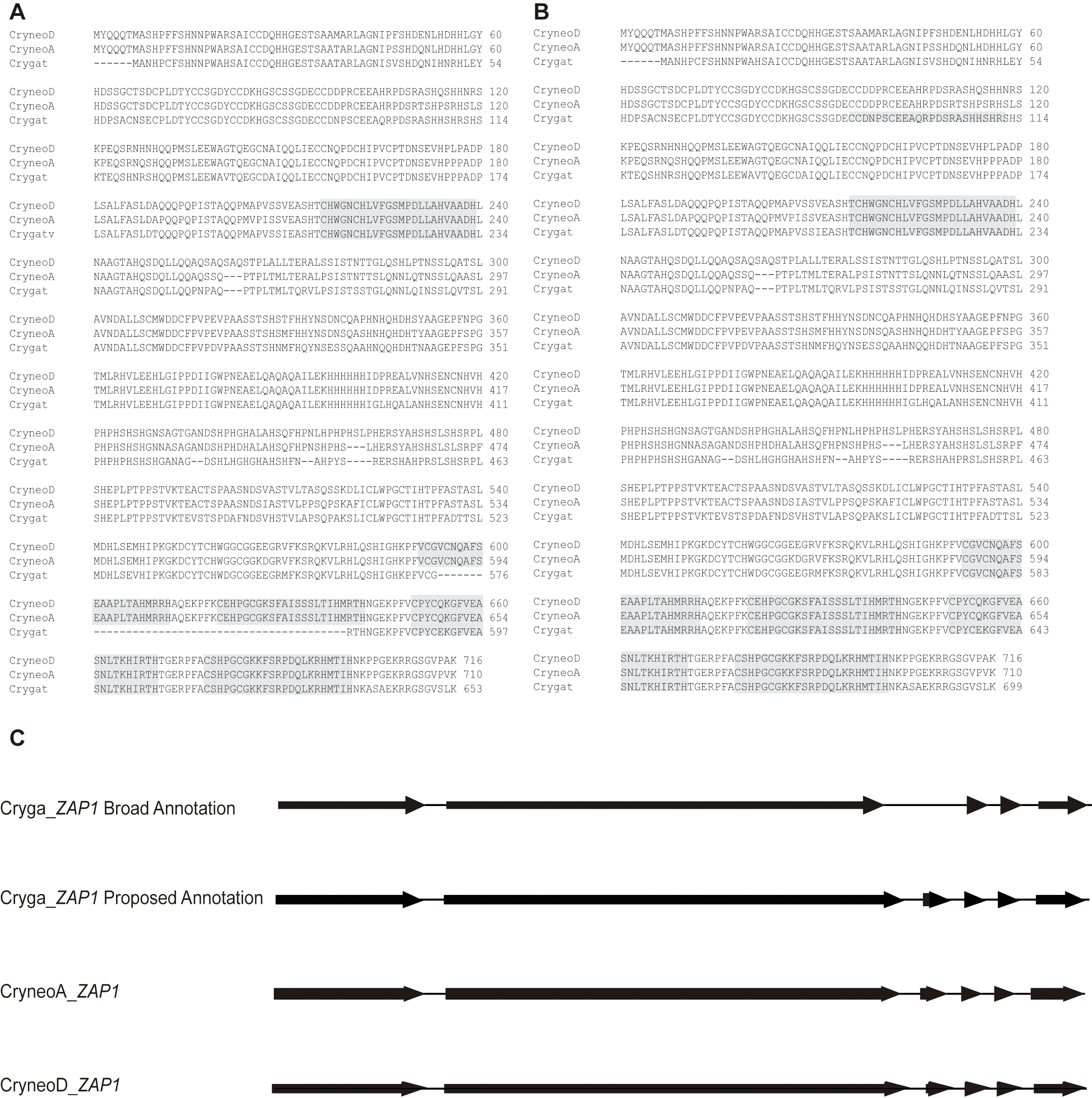

Supplement: Figure S1 — In silico characterization of C. gattii Zap1. Multiple sequence alignment of the Zap1 ortholog sequences of C. neoformans serotype D Zap1 (CryneoD_Zap1 – Genbank XP_572252) and C. neoformans serotype A Zap1 (CryneoA_Zap1 – Broad Institute CNAG_05392) with the C. gattii Zap1 Broad Institute (A) and the proposed sequences (B). The predicted zinc fingers are shown as shaded black boxes. (C) Comparison of the CNBG_4460 locus (Supercontig 11: 588643–591042) automatic annotation and the proposed annotation with the annotated sequences of ZAP1 from C. neoformans serotype D (CryneoD_ZAP1 – Genbank NC_006693) and C. neoformans serotype A (CryneoA_ZAP1 – Broad Institute CNAG_05392). Exons are depicted with arrows and arrowheads. (TIF) [file pone.0043773.s001.tif]

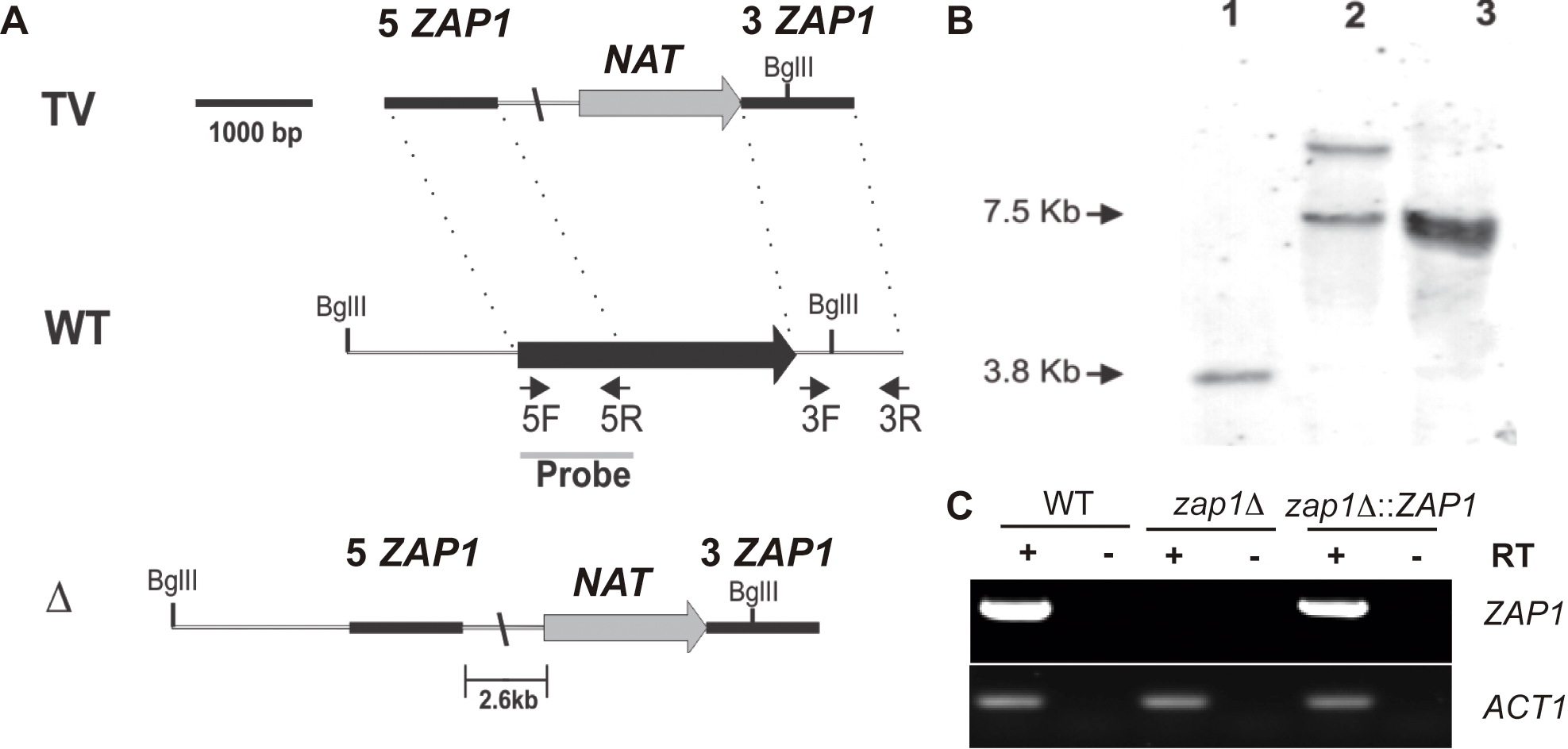

Supplement: Figure S2 — Construction of the C. gattii ZAP1 gene knockout and complemented strains. A. ZAP1 deletion scheme. TV represents the targeting vector constructed by Delsgate methodology. 5 ZAP1 and 3 ZAP1 represent the 5′ and 3′ flanking regions of the ZAP1 gene, respectively. 5F and 5R: primers utilized to amplify the 5′ flanking region of ZAP1. 3F and 3R: primers utilized to amplify the 3′ flanking region of ZAP1. Nat: cassette that confers nourseothricin resistance. WT represents the wild type locus of the ZAP1 gene in the R265 strain. Δ represents the ZAP1 locus in the zap1 mutant strain. The cleavage sites of the BglII restriction enzyme are indicated. B. Confirmation by Southern blotting. Genomic DNA (10 μg) from WT (lane 1), zap1Δ::ZAP1 complemented (lane 2) and zap1Δ mutant (lane 3) strains was digested with BglII. The 3′ flanking region was used as the probe for Southern hybridization. Numbers on the left indicate the hybridization signal sizes based upon the position of the molecular size marker. C. Semi-quantitative RT-PCR using cDNA from WT, zap1Δ mutant and zap1Δ::ZAP1 complemented strains as the template. RNA samples were used as templates for reactions employing (+) reverse transcriptase. Control reactions without reverse transcriptase addition (−) were used to confirm the absence of genomic DNA. The upper panel shows the ZAP1 amplicons, while the lower panel shows the ACT1 amplicons (loading control). (TIF) [file pone.0043773.s002.tif]

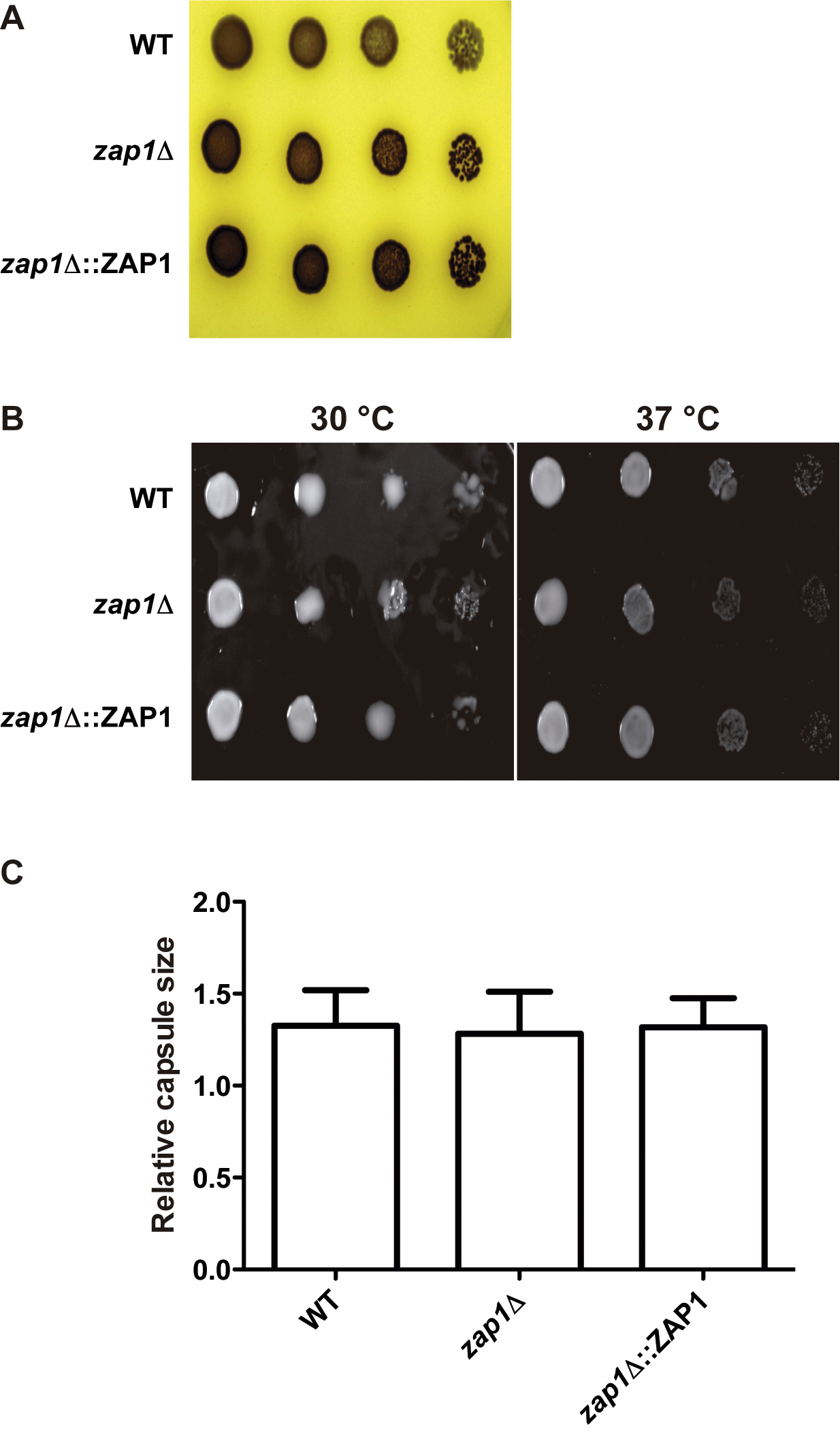

Supplement: Figure S3 — Analysis of virulence-related phenotypes of the C. gattii ZAP1 null mutant. (A) Melanin production was assessed by plating ten-fold serial dilutions of WT, zap1Δ mutant and zap1Δ::ZAP1 complemented strains in niger seed agar and incubating for 48 h. (B) Ability to replicate at body temperature was assessed by plating ten-fold serial dilutions of WT, zap1Δ mutant and zap1Δ::ZAP1 complemented strains in YNB agar and incubating at 30 or 37°C for 24 h. (C) Capsule production was evaluated by analysis of the capsule/cell ratio of 100 distinct cells from WT, zap1Δ mutant and zap1Δ::ZAP1 cells cultured in capsule-inducing conditions. (TIF) [file pone.0043773.s003.tif]
