# Supplementary material for: Zap1 Regulates Zinc Homeostasis and Modulates Virulence in Cryptococcus gattii
Source: PLoS One. 2012 Aug 20;7(8):e43773. doi: 10.1371/journal.pone.0043773 (PMC3423376; doi:10.1371/journal.pone.0043773)
Supplement: Table S2 — List of primers used in this work. (DOCX) [file pone.0043773.s005.docx]

**Supplementary Table 1: List of primers used in this work.**

| **Primer name** | **Sequence (5’-3’)** | | **Purpose** | |
| --- | --- | --- | --- | --- |
| ZAP1-5F | | AAAATAGGGATAACAGGGTAATCAGCCACAACAATCCTTGGG | 5-flank amplicon of *ZAP1* knockout construct / RT-PCR |  |
| ZAP1-5R | | GGGGACAAGTTTGTACAAAAAAAGCAGGCTATATCCCACATGCACGATAGTA | 5-flank amplicon of *ZAP1* knockout construct / RT-PCR |  |
| ZAP1-3F | | GGGGACCACTTTGTACAAGAAAGCTGGGTAGCGGTAAGAAATTTTCACGT | 3-flank amplicon of *ZAP1* knockout construct |  |
| ZAP1-3R | | AAAAATTACCCTGTTATCCCTAGCAACTTGAGACCGTCCCCT | 3-flank amplicon of *ZAP1* knockout construct |  |
| ZAP1compF | | ACAGCTATGACCATGATTACTTTTCTATATGCAACAAACTAGTCG | *ZAP1* amplicon for complementation |  |
| ZAP1compR | | GCACACTGGCGGCCGTTACTAATAAATTGATTACACGA | *ZAP1* amplicon for complementation |  |
| CGACTF | | CGGTATCGTCACAAACTGG | Amplification of *ACT1* for RT-PCR/qRT-PCR |  |
| CGACTR | | GGAGCCTCGGTAAGAAGAAC | Amplification of *ACT1* for RT-PCR/qRT-PCR |  |
| RTCgZAP1F | | GTGGCGAAGAAGGGAGAATG | Amplification of *ZAP1* for qRT-PCR |  |
| RTCgZAP1R | | ACAGTACGGGCAGACAAATGG | Amplification of *ZAP1* for qRT-PCR |  |
| CNBG5153R | | CAACATCGCTAACCTTAGTGC | Amplification of *GPX2* for qRT-PCR |  |
| CNBG5153F | | GAGTGACTTTCCCTATTGCC | Amplification of *GPX2* for qRT-PCR |  |
| CNBG4202F | | GAACGAGGTCTTTGCTTG | Amplification of *GPX1* for qRT-PCR |  |
| CNBG4202R | | GCTTCTCTGGCTTAGTGG | Amplification of *GPX1* for qRT-PCR |  |
| CNBG4667F | | ACTTCCGACTCTGCTGATTC | Amplification of *CAT3* for qRT-PCR |  |
| CNBG4667R | | GCTGAAAGGGGTAGATTCC | Amplification of *CAT3* for qRT-PCR |  |
| CNBG5786F | | CGTCAGTGAGGTTTATGCG | Amplification of *CAT2* for qRT-PCR |  |
| CNBG5786R | | GATGTGTATGCCGTAACTCG | Amplification of *CAT2* for qRT-PCR |  |
| CNBG4696F | | TCACTACTGCGAAGGTTTTG | Amplification of *CAT1* for qRT-PCR |  |
| CNBG4696R | | AAGACTGGGATGTTGTTGC | Amplification of *CAT1* for qRT-PCR |  |
| CNBG0599F | | TCCCGTTACTGTTTCTGG | Amplification of Cu/Zn SOD transcript for qRT-PCR |  |
| CNBG0599R | | GTTGCCGTCAGTCTTGAC | Amplification of Cu/Zn SOD transcript for qRT-PCR |  |
| CNBG2661F | | CCTTCCCCGAACTACTCTC | Amplification of Mn-SOD transcript for qRT-PCR |  |
| CNBG2661R | | CGTAAGTCTGGTGATGCTTG | Amplification of Mn- SOD transcript for qRT-PCR |  |
| CNBG5361F | | GTGTAGCCCTTCTTTTCTCC | Amplification of *ZIP3* for qRT-PCR |  |
| CNBG5361R | | CAATCCTGTCCGTTGGTAC | Amplification of *ZIP3* for qRT-PCR |  |
| CNBG6066F | | GCTGAAGTCGCCGCTTATC | Amplification of *ZIP1* for qRT-PCR |  |
| CNBG6066R | | GGGAGGGATGGATGTGATG | Amplification of *ZIP1* for qRT-PCR |  |
| CNBG2209F | | GTCATATTGGGCAAACTGG | Amplification of *ZIP2* for qRT-PCR |  |
| CNBG2209R | | AGGGGCAACAGACTCATAG | Amplification of *ZIP2* for qRT-PCR |  |
